# Editorial: Arrhythmias in women

**DOI:** 10.3389/fcvm.2024.1348878

**Published:** 2024-02-13

**Authors:** Hector Barajas-Martinez, Michael Shehata, Yang Liu

**Affiliations:** ^1^ Lankenau Institute for Medical Research, Lankenau Heart Institute, Wynnwood, PA, United States; ^2^ Cedars Sinai Heart Institute, Los Angeles, CA, United States; ^3^ Guangdong Cardiovascular Institute, Guangdong Provincial People's Hospital, Guangdong Academy of Medical Sciences, Southern Medical University, Guangzhou, China

**Keywords:** arrhythmia, gender difference, women, atrial fibrillation, sudden cardiac arrest

**Editorial on the Research Topic** Arrhythmias in women

Gender differences exist in cellular and clinical cardiac electrophysiology. Sex hormones might contribute to these disparities which could affect the epidemiology, presentation, clinical course, therapy response and prognosis of various arrhythmias in women and men. There is obvious gender-specific phenotypic heterogeneity in inherited arrhythmias. Female gender is associated with a higher risk of lethal cardiac events among patients with congenital long QT syndrome. In contrast, female is less prevalent in Brugada syndrome and has less frequent cardiac events. Women are also much easier to have QT intervals prolongation in response to antiarrhythmic drugs, suffer anecdotally more procedural complications during catheter ablation or implantable device therapy for arrhythmias, and have a higher risk of stroke when atrial fibrillation occurs. Additionally, there is an increased risk of arrhythmias during pregnancy and conventional treatment strategies could be largely limited in this scenario.

Understanding and awareness of important gender-specific pathophysiologic differences may help facilitate arrhythmia diagnosis and risk stratification as well as provide appropriate therapeutic decisions in women, thus improving their clinical outcomes. The Research Topic “Arrhythmias in Women”, with two original articles, one case report and one systematic review and meta-analysis, covered some aspects of sex-associated differences in clinical practice, including out-of-hospital cardiac arrest (OHCA) with refractory ventricular arrhythmias (VAs), left atrial substrate in patients with persistent atrial fibrillation (AF), catheter ablation of ventricular premature contractions (PVCs) in pregnancy and atrial fibrillation in breast cancer.

The study of Caputo et al. aimed to characterize sex-related differences in clinical presentation, cardiovascular risk profile, prevalence of coronary artery disease, and outcome in victims of OHCA presenting with refractory VAs which were defined as ventricular fibrillation or unstable ventricular tachycardia persisting despite three shocks. A total of 680 OHCA patients with initial shockable rhythm were included in this study and 216 had refractory VAs. It was demonstrated that OHCA presenting with refractory VAs was more frequently observed in male patients with a male-to-female ratio of 5:1. The refractoriness of arrhythmic events in males was probably associated with cardiac risk factors, especially preexisting coronary artery disease. Female patients with refractory VAs were relatively uncommon and no definite relation to a previous history of coronary artery disease was observed in their study. This sex-related difference implies potential etiologic heterogeneity for such entity among different genders and warrants further studies to understand the sex-specific mechanisms.

In the work of Marzak et al., they assessed the gender-related differences in left atrial bipolar voltage and low-voltage zones in patients with persistent AF, using a three-dimensional electro-anatomical mapping in sinus rhythm before catheter ablation. They found that the left atrial bipolar voltage amplitude was significantly lower in women than in men after 60 years of age. The female patients had a higher incidence and a greater extent of low-voltage zones. Furthermore, the low-voltage zones occurred earlier in women compared with men. These findings imply the presence of gender-specific atrial remodeling and might partially explain why female patients more frequently suffer arrhythmia recurrences after AF ablation. A substrate-based ablation strategy for women with AF appears to be encouraged according to this study, however, randomized studies on comparing ablation with and without voltage-guided strategies are still needed to validate its effects on women prognosis.

Li et al. reported a pregnant woman at 12 weeks with a high burden of PVCs exceeding 20% who experienced successfully zero x-rays radiofrequency ablation. According to the surface ECG, the PVCs were speculated to originate in the left coronary cusp. Activation mapping of PVCs using a three-dimensional electro-anatomical system identified the origin. Radiofrequency energy delivered in the left coronary cusp and eliminated the PVCs immediately. Traditionally, fluoroscopy is the primary tool for visualizing cardiac catheter position. However, it is largely limited for pregnant women because of potential teratogenic effects on fetus. The effects are directly related to the level of radiation exposure and stage of fetal development. This case report demonstrated that catheter ablation for PVCs with zero or minimal fluoroscopy during pregnancy was safe and feasible. However, it is noteworthy that PVCs in the present case were located at the typical position which was relatively easy to reach for the ablation catheter with no need of complex manipulation.

Breast cancer is the most common cancer among women. The study of Yao et al. investigated the potential relationship between AF and breast cancer by conducting a systematic review and meta-analysis. Twenty-three studies with 8,537,551 individuals were included in their analysis. The prevalence and incidence of AF among patients with breast cancer was 3% and 2.7%, respectively. Low certainty evidence suggested that breast cancer might increase the risk of AF, while moderate certainty evidence demonstrated that AF might be associated with increased risk of breast cancer. Yao et al. finally concluded that there was a bidirectional association between AF and breast cancer. The reported prevalence of AF among patients with breast cancer in the present study seems to be not significantly higher than that in general population. A causal relationship therefore still needs to be proved in further studies.

In summary, the current Research Topic for Frontiers in Cardiovascular Medicine provides the readers with some special content about arrhythmias in women. Female patients demonstrate distinctive clinical features in etiologies of malignant VAs and atrial substrate related to persistent AF. Management of arrhythmias for pregnant women is still challenging. Zero and minimal fluoroscopic approaches during catheter ablation provide a valuable alternative to the management of arrhythmias. Emerging evidence suggests common mechanisms between cancer and AF. Further studies of the common underlying pathophysiology of female-specific cancer and arrhythmia are warranted and may lead to novel therapeutic and diagnostic strategies to improve the care and prognosis of this growing patient population.

